# Attention – oscillations and neuropharmacology

**DOI:** 10.1111/j.1460-9568.2009.06833.x

**Published:** 2009-08

**Authors:** Gustavo Deco, Alexander Thiele

**Affiliations:** 1 Computational Neuroscience Group, Department of Technology, Universitat Pompeu FabraBarcelona, Spain; 2 Institute of Neuroscience, Newcastle UniversityNewcastle upon Tyne NE2 4HH, UK

**Keywords:** acetylcholine, macaque monkey, modelling, neuronal coherence, *N*-methyl-d-aspartate, spatial integration

## Abstract

Attention is a rich psychological and neurobiological construct that influences almost all aspects of cognitive behaviour. It enables enhanced processing of behaviourally relevant stimuli at the expense of irrelevant stimuli. At the cellular level, rhythmic synchronization at local and long-range spatial scales complements the attention-induced firing rate changes of neurons. The former is hypothesized to enable efficient communication between neuronal ensembles tuned to spatial and featural aspects of the attended stimulus. Recent modelling studies suggest that the rhythmic synchronization in the gamma range may be mediated by a fine balance between *N*-methyl-d-aspartate and α-amino-3-hydroxy-5-methylisoxazole-4-propionate postsynaptic currents, whereas other studies have highlighted the possible contribution of the neuromodulator acetylcholine. This review summarizes some recent modelling and experimental studies investigating mechanisms of attention in sensory areas and discusses possibilities of how glutamatergic and cholinergic systems could contribute to increased processing abilities at the cellular and network level during states of top-down attention.

## Introduction

Attention is necessary for all cognitive tasks, tasks that in their most simple form require stimulus selection, response selection and performance monitoring. It serves to tune out irrelevant information, enhance perceptual abilities and increase our readiness to respond appropriately. This is achieved by increased sensory representation of the attended objects at the neuronal level, which has been demonstrated for most visual cortical areas by means of single-unit recordings in macaque monkeys (Moran & Desimone, 1985; Spitzer *et al.*, 1988; Motter, 1993; Treue & Maunsell, 1996; Roelfsema *et al.*, 1998; Reynolds *et al.*, 1999; Roberts *et al.*, 2007) and by means of increased blood oxygenation level-dependent signals using functional magnetic resonance imaging in humans (Kastner *et al.*, 1998; Tootell *et al.*, 1998; Gandhi *et al.*, 1999; Kastner & Ungerleider, 2000; Silver *et al.*, 2005, 2007).

To understand how attention is implemented in the brain it is important to distinguish the control signals responsible for the generation and maintenance of attention (which originate in higher ‘source’ areas) from their effect on sensory processing and perception (which are visible in sensory areas as described in some detail below). Studies in humans and macaque monkeys have demonstrated that the control signals are mediated and maintained within circumscribed networks of the parietal and frontal cortex (e.g. Steinmetz & Constantinidis, 1995; Duncan *et al.*, 1997; Thompson & Schall, 1999; Kusunoki *et al.*, 2000; Everling *et al.*, 2002, 2006; Shulman *et al.*, 2002; Bisley & Goldberg, 2003; Mayer *et al.*, 2004; Constantinidis & Steinmetz, 2005; Fan *et al.*, 2005; Kincade *et al.*, 2005). These areas control attentional selection and influence sensory processing and perception via feedback projections (Desimone & Duncan, 1995; Luck *et al.*, 1997), as demonstrated by microstimulation studies (Moore & Armstrong, 2003; Armstrong & Moore, 2007; Moore & Fallah, 2004; Armstrong *et al.*, 2006; Ekstrom *et al.*, 2008) and by simultaneous recordings in sensory and source areas (Gregoriou *et al.*, 2009). Although there are many open questions and possible scenarios regarding the implementation of feedback projections, here we will treat their existence as a ‘given’. Our focus will be on how attention affects neuronal network constellations in sensory areas to selectively alter activity level and pattern, possible mechanisms of its implementation and the contribution of selected neurotransmitters.

## Attention and biased competition

Although a multitude of studies have demonstrated the effects of attention on basic stimulus processing, an experimental protocol used by Reynolds *et al.* (1999) allowed the explicit separation of sensory processing mechanisms from attentional effects. They demonstrated that the response of a V4 neuron to two stimuli in its receptive field in the absence of any attentional influences is not the sum of its responses to each stimulus alone. If one of the stimuli was an effective stimulus, whereas the other was an ineffective stimulus, the response to both stimuli presented simultaneously was a weighted average of the responses to each stimulus alone. Importantly, if spatial attention was allocated to the effective stimulus the neuronal activity was up-regulated towards the level of response corresponding to the effective stimulus alone. However, if spatial attention was allocated to the ineffective stimulus, then the neuronal activity was down-regulated towards the level of response elicited by the ineffective stimulus alone. Thus, attentional selection appears to operate by biasing an existing competitive interaction between multiple stimuli in the visual field toward one stimulus or another, so that behaviourally relevant stimuli are processed in the cortex, whereas irrelevant stimuli are filtered out (Duncan & Humphreys, 1989; Desimone & Duncan, 1995; Duncan & Nimmo-Smith, 1996).

## Modelling biased competition

The synaptic and spiking mechanisms that may underlie biased competition have been analysed in a variety of different network models. Deco & Rolls (2005) have implemented a model of cortical areas V2 and V4 that exhibits biased competition signatures. Within this model both areas have the same internal architecture, with dynamic competition between neurons of different feature selectivity. Neurons were modelled as integrate and fire neurons with realistic synaptic *N*-methyl-d-aspartate (NMDA), α-amino-3-hydroxy-5-methylisoxazole-4-propionate (AMPA) and γ-aminobutyric acid receptor dynamics (see Deco & Rolls, 2005 for details). Provided that the model is implemented with a particular connectivity, it generates dynamics that are consistent with the experimental results of biased competition (Reynolds *et al.*, 1999), i.e. the dynamics show the level of competition and attentional biased up- and down-regulation observed experimentally in V2 and V4. Biased competition can be modelled in a variety of slightly different network configurations. Ardid *et al.* (2007) generated a reciprocally connected loop of a sensory and a working memory network comprised of biophysically-based spiking excitatory and inhibitory neurons. In this model the working memory module exhibited strong recurrent excitation, whereas the sensory network was dominated by inhibition. This model was able to replicate multiplicative gain changes, biased competition and feature similarity gain control, all of which have been reported to occur as a function of attention. Yet others have used modified versions of predictive coding to model biased competition in visual attention (Hamker, 2005; Spratling, 2008). A common feature of all of these models is that through dynamic competition a slight additional external input in one of the neuronal populations (bottom-up or top-down mediated) is amplified, whereas the activities of the other populations are partially suppressed, implementing in this way an effective filtering mechanism.

## The role of neuronal synchrony: gamma band modulation

Oscillations in the gamma frequency band (30–100 Hz) have been found in most species and brain areas investigated, including the visual cortex (Gray *et al.*, 1989; de Oliveira *et al.*, 1997). Synchronization of neuronal activity in the gamma frequency band has been shown to be involved in several fundamental functions in the brain. Notably, neurons selected by attentional mechanisms show enhanced synchronization (Steinmetz *et al.*, 2000), which is particularly prominent in the gamma band (Fries *et al.*, 2001). Fries *et al.* (2001) recorded activity from area V4 while macaque monkeys were attending behaviourally relevant stimuli. They found that neurons activated by the attended stimulus showed increased gamma frequency synchronization compared with neurons activated by the distractor. A variety of different models have explored how synchrony in a network can be modulated in a manner similar to what is seen in V4 in attention-demanding tasks. Changes in synchrony occur in competing cortical interneuron networks (Tiesinga & Sejnowski, 2004) and attention-mediated increases in neuronal synchrony in the gamma range can be modelled by reducing adaptation currents in principal cells possibly through cholinergic mechanisms (Buhl *et al.*, 1998; Borgers *et al.*, 2005). Alternatively it can be mediated by decreasing the activity in inhibitory interneurons (Buia & Tiesinga, 2006), thereby releasing pyramidal cells from a ‘bath of inhibition’ (Borgers *et al.*, 2008). The reduction of inhibitory drive on pyramidal cells might be mediated by cholinergic activation of interneuron–interneuron inhibition (Borgers *et al.*, 2008). However, a cascade of interneuron interactions may not be necessary, as acetylcholine (ACh) can directly inactivate specific interneurons (Xiang *et al.*, 1998).

**Fig. 1:**
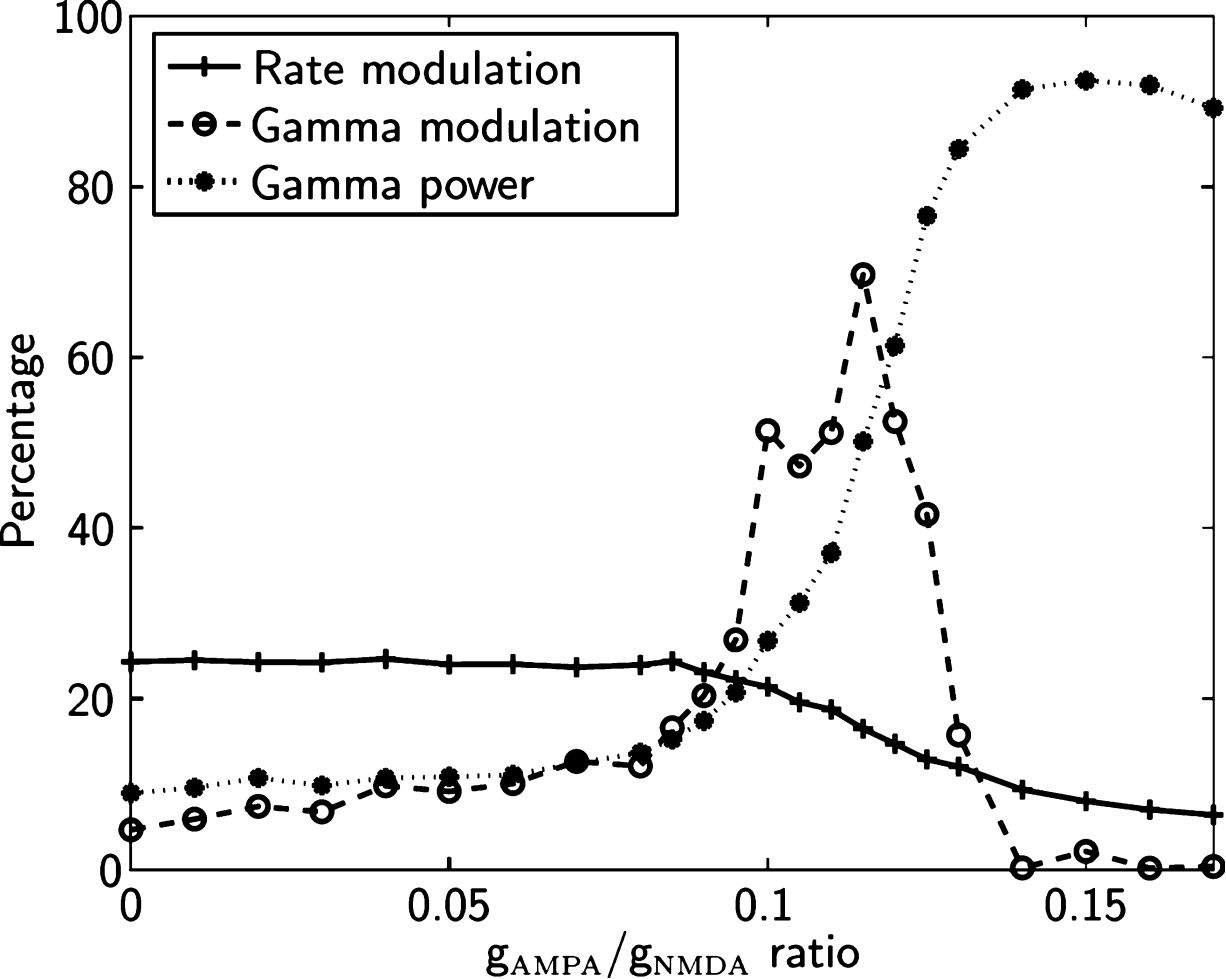
Attention-induced rate modulation (solid line) and gamma modulation (dashed line) as a function of the g_AMPA_:g_NMDA_ ratio in a network model. The initial main effect of increasing the g_AMPA_:g_NMDA_ ratio (with or without attention) was an increase in the network synchronization in the gamma band (dotted line), i.e. in the overall gamma power. The rate modulation decreased monotonically with the g_AMPA_:g_NMDA_ ratio. Attention-mediated gamma modulation (irrespective of overall power) increased to a g_AMPA_:g_NMDA_ ratio of 0.12 and then decreased quickly to almost 0. Attention-induced modulation of gamma synchrony depended on a fine balance of NMDA/AMPA receptor activation, whereas attention-induced rate changes were less susceptible to small changes in this ratio. The *x*-axis shows the ratio of AMPA:NMDA conductance (for details see Buehlmann & Deco, 2008) and the *y*-axis shows the percentage of modulation of gamma band frequency for the rate and gamma modulation data; it shows the percentage of gamma power relative to overall spectral power for the gamma power curve.

All of the above models provide possible solutions as to how attention-mediated changes in neuronal synchrony might be implemented at the neuronal level but they do not address the question of whether synchrony plays a fundamental role in cognition. An attempt to provide some insight into this controversial topic was made by Buehlmann & Deco (2008) who investigated whether neuronal correlates of attention are rate-based (as demonstrated in biased competition) or oscillation-based (as demonstrated in the studies of Fries *et al.*, 2001). They used the same network model with which biased competition had been studied (Deco & Rolls, 2005) but now delineated the parameters that allow for increased oscillation in the gamma range between neurons representing the attended location. Oscillations can be generated by a pyramidal-to-interneuron loop (Whittington *et al.*, 1995; Traub *et al.*, 1996; Brunel & Wang, 2003; Whittington & Traub, 2003; Gieselmann & Thiele, 2008), whereby the oscillation frequency depends on the relative timescales of the excitatory and inhibitory decay constants. Faster excitation than inhibition, or a higher excitation:inhibition ratio, favours the feedback loop and gives rise to oscillations in the gamma range (Brunel & Wang, 2003). In the network of Buehlmann & Deco (2008), oscillations were manipulated by adjusting the conductances of AMPA and NMDA receptors (g_AMPA_ and g_NMDA_). An increase of g_AMPA_ and a decrease of g_NMDA_ allowed the pyramidal-to-interneuron loop interactions to oscillate more strongly and resulted in increased gamma activity. Within this network it was possible to adjust the conductances of AMPA and NMDA receptors (and thus the excitation/inhibition recurrent cycle) such that the network only showed gamma oscillations during stimulus presentation. Interestingly, these changes in the AMPA and NMDA receptor conductance of the network also determined whether attentional bias affected the rates more strongly or had a larger effect on the gamma synchronization (Buehlmann & Deco, 2008). This result is plotted in Fig. 1. It showed that rate modulation and gamma modulation in the network were not concomitant phenomena. Attentional rate modulation occurred for a broad range of the g_AMPA_:g_NMDA_ ratios, whereas attentional modulation of the gamma band activity was restricted to a relatively narrow range of g_AMPA_:g_NMDA_ ratios.

## Behavioural consequences of increased gamma synchronization

Attention-induced increases of gamma band modulation thus occur *in vivo* and *in silico.* The crucial question remains as to whether such changes have behavioural relevance. Additional analyses of the original data set of Fries *et al.* (2001) revealed that stronger gamma band modulations in V4 correlated with faster reaction times (RTs) of the monkeys (Womelsdorf *et al.*, 2006). Equivalent results could be found in the network model of Buehlmann & Deco (2008). Their proxy of RT was firing rate-based (i.e. a neuronal RT). They analysed the time that it takes the stimulated neuron pool to rise to its mean firing frequency. Overall they found that neuronal RTs were different for the attended and unattended neuron pool, with attended pools exhibiting faster RTs. Importantly, they also found that it was necessary to keep the g_AMPA_:g_NMDA_ conductance ratio within a fairly narrow range to produce the following four key results. (i) This narrow range of g_AMPA_:g_NMDA_ conductance ratios generated the overall fastest neuronal RTs. (ii) It generated the largest RT differences between the attended and unattended pool. (iii) It allowed attention to strongly increase gamma modulations. (iv) Neuronal RTs inversely correlated with the attentional modulation in the gamma band, i.e. stronger attentional gamma band modulation led to shorter neuronal RTs. Thus, *in-vivo* (Womelsdorf *et al.*, 2006) and *in-silico* (Buehlmann & Deco, 2008) results suggest a behavioural role for gamma synchronization during attentional selection.

## The role of acetylcholine

In addition to feedback, the neuromodulator ACh, originating in the basal forebrain (Marrocco *et al.*, 1994; Voytko *et al.*, 1994; Voytko, 1996; Robbins, 1997; Witte *et al.*, 1997; Sarter *et al.*, 1999, 2001; Gill *et al.*, 2000; Dalley *et al.*, 2001), is likely to contribute to attention. Although cholinergic modulation of cortical processes can be signal driven, it can equally be top-down driven (Sarter *et al.*, 2005; Parikh *et al.*, 2007). Top-down-driven modulation of the corticopetal cholinergic system is under the control of ‘executive’ areas in the prefrontal cortex, thereby influencing their own ACh levels, those in parietal (Nelson *et al.*, 2005) and sensory cortical areas. This form of top-down control enhances the cognitive modulation of detection processes (Nelson *et al.*, 2005). Within this framework, the ascending cholinergic system can be conceptualized as part of the top-down control system emanating within the prefrontal cortex by which control is exerted over processing resources in the parietal attention system and in sensory cortices (Sarter *et al.*, 2005). The cholinergic input to the cortex consists of modules that specifically target selected cortical areas in a task- and context-dependent manner (Zaborszky & Duque, 2000; Zaborszky, 2002). Complementary studies show that ACh release in the frontal cortex closely follows the time-course of attention-demanding events (Parikh *et al.*, 2007) and behavioural studies in humans suggest that ACh (by means of muscarinic receptors) is responsible for the maintenance of attention but not for attention switching (Furey *et al.*, 2008). Notably, increased levels of ACh are related to attentional effort, rather than attentional performance (Kozak *et al.*, 2006).

Despite this wealth of data, it is unclear how ACh contributes to neuronal processing during selective attention. What are the cellular mechanisms by which ACh enables and boosts different aspect of attentional processing? Does ACh affect firing rates, does it affect synchrony, or both? Despite a lack of direct evidence for some of these questions, predictions can be based on slice studies, data from anaesthetized animals, as well as recent data in task-performing macaques. Early reports from cat V1 suggested that ACh increases the signal-to-noise ratio (Sillito & Kemp, 1983; Sato *et al.*, 1987), which could be a mechanism by which attention acts. However, recordings in primate V1 failed to replicate this (Zinke *et al.*, 2006). ACh reduces spike frequency adaptation, thus possibly mediating increased stability of stimulus or abstract representations at the single neuron level (McCormick & Prince, 1985, 1986), which would benefit attentional processing. It is tempting to speculate that ACh increases the representation of stimuli at the current attentional focus, and protects it against interference from competing stimuli, thus resulting in reduced distractibility. Thereby task-relevant information would be processed more effectively, which comes at the expense of irrelevant information. Recent data from our laboratory in the anaesthetized and awake primate are supportive of this idea. Based on the *in-vitro* studies we argued that ACh might alter the flow of feedforward and lateral/feedback information in the cortex. ACh suppresses the efficacy of intracortical synapses by activating muscarinic receptors (Hasselmo & Bower, 1992; Hasselmo, 1995; Kimura & Baughman, 1997; Kimura *et al.*, 1999; Hsieh *et al.*, 2000; Linster & Hasselmo, 2001) and increases the efficacy of feedforward/thalamocortical input (Gil *et al.*, 1997) by acting on presynaptic nicotinic receptors located on thalamocortical synapses (Prusky *et al.*, 1987). In the primary visual cortex, thalamocortical inputs are largely responsible for classical receptive field response properties (Dragoi *et al.*, 2000), whereas non-classical receptive field influences are mostly mediated by lateral and feedback connections (Das & Gilbert, 1999; Angelucci *et al.*, 2002; Cavanaugh *et al.*, 2002). Thus, ACh applied *in vivo* should reduce the impact of stimuli presented in the non-classical receptive field while increasing the effect of stimuli placed within the classical receptive field. This proposal was investigated in V1 of anaesthetized marmoset monkeys (Roberts *et al.*, 2005) by measuring the neuronal spatial integration properties. Roberts *et al.* (2005) predicted that spatial summation should be reduced when ACh was externally applied compared with when it was not applied. In line with this prediction, Roberts *et al.* (2005) found that the application of ACh resulted in a reduction of spatial integration, which was mediated by a reduction of the spatial summation area.

If ACh contributes to attentional modulation, then attention should reduce spatial integration at the perceptual and neuronal level. A variety of human psychophysical studies have indeed demonstrated that selective spatial attention can ensure that task-irrelevant information (information surrounding a target) has a reduced influence on perception. In these studies attention served to reduce the influence of behaviourally irrelevant contextual stimuli (Ito *et al.*, 1998; Zenger *et al.*, 2000; Roberts & Thiele, 2008) or served as a noise-exclusion mechanism when stimuli had to be detected in noisy environments (Lu & Dosher, 1998; Dosher & Lu, 2000; Lu *et al.*, 2002). In line with the above prediction, Roberts *et al.* (2007) reported that attention reduced the spatial integration of parafoveal cells in V1 and the underlying changes were mediated by a change in the spatial summation area (Roberts *et al.*, 2007). Similar results have recently been reported for V4. Attention strongly affects centre-surround interactions of V4 neurons (Sundberg *et al.*, 2009), ensuring that distractive influences by irrelevant stimuli are filtered out.

**Fig. 2:**
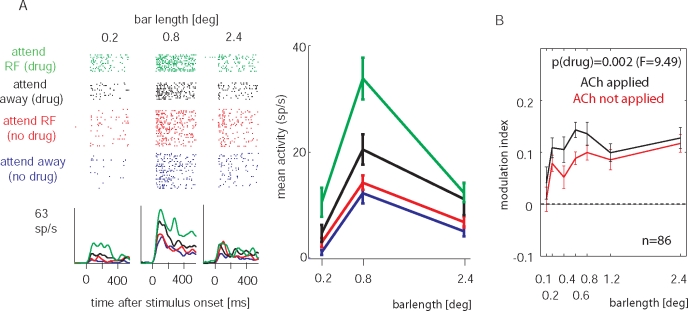
Influence of ACh on attentional modulation in V1. (A) Example of a single cell where the application of ACh resulted in enhanced attentional modulation. In the absence of ACh application the attentional modulation was small but significant (*P* < 0.05). Left plot shows single trial activity for the four conditions and the corresponding peri-stimulus time histograms. Right plot shows mean activity obtained from the time period of 200–500 ms after stimulus onset. (B) Mean attentional modulation index for our population of V1 cells. Attentional modulation was significantly increased when ACh was applied. RF, receptive field.

A direct test of the proposal that ACh is part of the machinery of attentional modulation in visual cortex has been performed recently (Herrero *et al.*, 2008). These authors combined iontophoretic pharmacological analysis of cholinergic receptors with single-cell recordings in V1 while rhesus macaque monkeys performed a task that demanded top-down spatial attention. Herrero *et al.* (2008) demonstrated that local ACh application significantly increased attentional modulation in V1 neurons (Fig. 2) and affected the animal’s behaviour. It was as if the animals had difficulties in disengaging attention from the location represented by the neurons that were influenced by ACh application. The effects of ACh on attentional modulation in V1 were mediated by muscarinic receptors. Interestingly, the nicotinic antagonist mecamylamine had no systematic effect on attentional modulation, whereas it reduced the overall neuronal gain. The latter was somewhat expected as nicotinic receptors in V1 are largely located on presynaptic thalamocortical terminals (Wonnacott, 1997) where nicotine increases the synaptic transmission efficacy (Disney *et al.*, 2007) but at the same time the thalamocortical synapse was unlikely to be the main site of attentional modulation in V1.

## Outlook

The data reviewed so far demonstrate that attention affects firing rates and neuronal synchrony in different cortical areas. Modelling work suggests that these two effects are complementary mechanisms. Attention-induced increases of gamma synchrony seem to depend on a finely tuned balance of NMDA/AMPA receptor activation. Attention-induced firing rate changes in V1 depend on cholinergic activation of muscarinic receptors. Intriguingly, increased cortical synchrony in the gamma band range can also be induced by stimulation of the mesencephalic reticular formation (Munk *et al.*, 1996), which triggers increased levels of ACh in the cortex (Szerb, 1967). Moreover, stimulation of the nucleus basalis, the main source of cortical ACh, results in EEG activation in the auditory cortex and it changes cellular membrane potential fluctuations from high-amplitude low-frequency to small-amplitude gamma frequency oscillations (Metherate *et al.*, 1992). Application of the muscarinic antagonist scopolamine in V1 of anaesthetized cats resulted in reductions of gamma oscillations but local application of ACh surprisingly failed to increase gamma power directly (Rodriguez *et al.*, 2004), although it affected gamma synchronization at longer timescales. It is thus tempting to speculate that ACh contributes to increased gamma oscillations as seen during selective attention in V4 (Fries *et al.*, 2001) in addition to the proposed balanced NMDA:AMPA activation ratio (Buehlmann & Deco, 2008). Future work on the mechanisms of attention might focus on delineating whether ACh contributes to changes in cortical synchrony during states of attention in addition to its contribution to firing rate changes (Herrero *et al.*, 2008). In addition, it will be important to investigate whether a change of the ratio of g_AMPA_:g_NMDA_ (e.g. by application of the NMDA antagonist 5-phosphono-dl-norvaline dl-2-amino-5-phosphonovaleric acid) will abolish attention-induced changes in gamma power as predicted by Buehlmann & Deco (2008). Notably, this modelling predicts that such manipulations should mostly affect the strengths of attention-induced gamma oscillations, whereas attention-induced rate modulations should be less strongly affected.

The possible contribution of NMDA receptors to the attention-mediated increase in gamma oscillation is intriguing, as cognitive disorders are often associated with abnormal neural synchronization (Uhlhaas & Singer, 2006) and in schizophrenia this seems partially related to NMDA receptor hypofunction (Lisman *et al.*, 2008). Despite this apparent link, the NMDA receptor hypofunction and its effect on gamma synchronization in schizophrenia are hypothesized to reduce excitation of fast-spiking interneurons (Lisman *et al.*, 2008), whereas NMDA receptor-mediated effects in the model were pyramidal cell based (Buehlmann & Deco, 2008). Should the model thus be modified to investigate how alternative NMDA receptor locations affect function? This may be the case but it is important to highlight that NMDA receptor contributions to gamma synchronization can vary greatly between areas (Roopun *et al.*, 2008). Thus, caution is necessary when attempting to apply findings from one specific structure to another structure. In this respect it may be useful to highlight the differences between areas in terms of neuromodulator susceptibility that rides on top of the well-established similarity of cortical structure across areas. The similarity of cortical structure between areas has led to the useful concept of the canonical microcircuit (Douglas & Martin, 2004) but differences in receptor distribution from area to area (e.g. Disney *et al.*, 2006; Disney & Aoki, 2008) could be viewed as a canonical microcircuit breaker, by which processing flexibility is achieved. Due to these differences in receptor distribution, location and density it will be important to obtain a detailed understanding of receptor distributions in different areas and their specific role in cognitive functions. For such an understanding, immunohistochemical, *in-vitro*, *in-vivo* and *in-silico* approaches need to be integrated.

There is additional evidence suggesting that NMDA receptors play a ‘special’ role in attention. A striking similarity exists between changes in neuronal gain that are induced by direct NMDA application and those induced by selective attention (Fox *et al.*, 1990; Williford & Maunsell, 2006; Thiele *et al.*, 2009). Recent work has suggested that feedback projections recruit synapses with NMDA:AMPA receptor ratios different from those of feedforward projections (Self *et al.*, 2008). Although direct evidence for this scenario is lacking, it is interesting to note that attention affects mostly the sustained part of visual responses in V1 (e.g. Roelfsema *et al.*, 1998) and a selective contribution of NMDA receptors to the sustained part of the response in the ventrobasal thalamus (Salt, 1986) has been demonstrated. In addition, slice studies have shown that cortico-cortical interactions between layer 4 cells are largely mediated by a variety of different NMDA receptors (Fleidervish *et al.*, 1998). Finally, studies in the primary and secondary motor cortex of the macaque demonstrate that NMDA and non-NMDA receptors contribute to different aspects and epochs of motor preparation and execution (Shima & Tanji, 1993). This evidence shows that synapses in the brain show functional specialization in terms of NMDA and non-NMDA receptor density. At the same time, the proposal that NMDA receptor-rich synapses are selectively recruited during attention is not predicted by either slice studies or modelling work. AMPA and not NMDA receptors play a dominant role in the generation of gamma oscillations of hippocampal and cortical slices (Buhl *et al.*, 1998; Fisahn *et al.*, 1998; LeBeau *et al.*, 2002). Modelling by Buehlmann & Deco (2008) predicts a specific ratio of AMPA:NMDA currents to be present in the ‘attend in’ and ‘attend away’ states, not necessarily attention-induced changes of this ratio. It will thus be interesting to study how neuronal gain is affected by attention when NMDA receptor antagonists are applied, as well as how this affects the ability of attention to selectively alter gamma local field potential power and spike field coherence.

It may also be interesting to determine the effects of NMDA receptor blockade on the frequency and overall strengths of gamma oscillations in the absence of attentional manipulations. In a pyramidal/interneuron network the frequency of oscillations is determined by the rise and decay time of the inhibitory and excitatory postsynaptic potentials (Brunel & Wang, 2003). Faster excitation than inhibition favours gamma frequency oscillations in the 30–80 Hz range and the ratio determines the frequency of oscillations (Brunel & Wang, 2003). Moreover, the ratio of pyramidal cell and interneuron contribution to the network drive affects the oscillation frequency (Brunel & Wang, 2003; Gieselmann & Thiele, 2008). Thus, in principle NMDA receptor activation should reduce the gamma oscillation frequency and gamma power. Conversely, NMDA receptor blockade should increase the gamma power and shift the dominant frequency towards higher values. If true, it might be worthwhile to investigate whether attention affects the frequency of gamma oscillation in the striate and extrastriate cortex, in addition to the overall power, as such effects might reveal some of the underlying receptor mechanisms.

The same theoretical and computational framework that has been used to study the contribution of NMDA and AMPA receptors can be extended for a detailed study of the modulatory effects of ACh in attention. Here it will be of interest to explore how ACh could mediate biased competition in a two-area network. It is likely that this will involve modifying the strength of the recurrent connections, strength of the feedforward (thalamocortical) input and level of Ca^2+^-dependent afterhyperpolarization adaptation. Parametric manipulation of these parameters in the model may reveal how each parameter contributes to attentional amplification and suppression in the biased competition network, and to what extent these changes are rate or synchrony based. If successful, the effect of cholinergic receptor agonist and antagonist on attention-mediated biased competition in V4 could be explored experimentally, in conjunction with possible NMDA receptor mechanism interactions.

Finally, it may be of interest to explore to what extent spatial attention and working memory are conceptually related (whereby top-down spatial attention is regarded as a spatial working memory signal), and to explore how these are related in terms of their dependence on cholinergic and NMDA mechanisms. Modelling suggests that persistent mnemonic activity can be mediated by synaptic reverberations in recurrent circuits enabled by NMDA receptors (Wang, 2001), whereas *in-vitro* studies showed that persistent memory-related activity in the entorhinal cortex depends on muscarinic receptor mechanisms (Egorov *et al.*, 2002).

The questions outlined in this review, although challenging, can now be addressed directly in task-performing monkeys. Such experiments will provide specific insights into the mechanisms of attention. The approaches are ideally informed by detailed model predictions but ultimately these are empirical questions that have to be tested experimentally. It is unlikely that all open questions can be resolved by probing attentional modulations in a single cortical area, as different areas show differences regarding neuromodulator receptor distributions (Mesulam *et al.*, 1983) and they also vary with respect to the receptor locations on specific cell types (Disney *et al.*, 2006; Disney & Aoki, 2008). Ultimately it will be necessary to study how different neurotransmitters contribute to cognitive function in many (if not all) cortical and subcortical areas, where the specific roles of different receptors will have to be delineated for each area. We also need to understand how the different transmitter systems contribute to rate and/or synchrony coding schemes if we want to understand cognition at the mechanistic level. Studies over the last 20 years have paved the way to perform this analysis within the framework of attention and we are now slowly heading towards such an understanding.

## Abbreviations

ACh: acetylcholine
AMPA: α-amino-3-hydroxy-5-methylisoxazole-4-propionate
g_AMPA_: conductance of α-amino-3-hydroxy-5-methylisoxazole-4-propionate receptors
g_NMDA_: conductance of *N*-methyl-d-aspartate receptors
NMDA: *N*-methyl-d-aspartate
RT: reaction time
